# Learning cardiac activation and repolarization times with operator learning

**DOI:** 10.1371/journal.pcbi.1013920

**Published:** 2026-01-27

**Authors:** Giovanni Ziarelli, Edoardo Centofanti, Nicola Parolini, Simone Scacchi, Marco Verani, Luca F. Pavarino

**Affiliations:** 1 Dipartimento di Matematica, Università di Milano, Milano, Italy; 2 Dipartimento di Matematica, Università di Pavia, Pavia, Italy; 3 MOX Laboratory - Dipartimento di Matematica, Politecnico di Milano, Milano, Italy; Nanjing University, CHINA

## Abstract

Solving partial or ordinary differential equation models in cardiac electrophysiology is a computationally demanding task, particularly when high-resolution meshes are required to capture the complex dynamics of the heart. Moreover, in clinical applications, it is essential to employ computational tools that provide only relevant information, ensuring clarity and ease of interpretation. In this work, we exploit two recently proposed operator learning approaches, namely Fourier Neural Operators (FNO) and Kernel Operator Learning (KOL), to learn the operator mapping the applied stimulus in the physical domain into the activation and repolarization time distributions. These data-driven methods are evaluated on synthetic 2D and 3D domains, as well as on a physiologically realistic left ventricle geometry. Notably, while the learned map between the applied current and activation time has its modeling counterpart in the Eikonal model, no equivalent partial differential equation (PDE) model is known for the map between the applied current and repolarization time. Our results demonstrate that both FNO and KOL approaches are robust to hyperparameter choices and computationally efficient compared to traditional PDE-based Monodomain models. These findings highlight the potential use of these surrogate operators to accelerate cardiac simulations and facilitate their clinical integration.

## Author summary

Cardiac electrophysiology simulations are crucial for understanding how electrical signals propagate through the heart. However, solving the underlying mathematical models—typically partial differential equations—requires significant computational resources, especially when high-resolution anatomical detail is involved. This limits their real-time use in clinical settings. In this study, we explore two operator learning techniques, Fourier Neural Operators (FNO) and Kernel Operator Learning (KOL), as efficient alternatives to traditional solvers. These machine learning models learn how to predict activation and repolarization times —the key quantities that describe the electrical behavior of the heart— directly from the input electrical stimulus. We test these models on idealized 2D and 3D domains, as well as on a realistic human left ventricle geometry. Remarkably, our learned models achieve high accuracy while being orders of magnitude faster than traditional solvers. While activation times can be related to well-established mathematical models, repolarization times lack such a direct modeling framework—making our data-driven approach especially valuable. Our findings suggest that operator learning methods can make high-fidelity cardiac simulations more accessible for clinical applications by drastically reducing computation time while maintaining accuracy.

## Introduction

Computational modeling of cardiac electrophysiology has become a fundamental tool for understanding heart function, diagnosing cardiac conditions, and developing therapeutic interventions [34,53]. Recent years have witnessed significant advances in both mathematical modeling, numerical techniques and computational capabilities, enabling increasingly sophisticated simulations of cardiac electrical activity [6,13,27,39]. Despite these advances, the computational complexity of high-fidelity cardiac models remains a substantial challenge, particularly for large-scale simulations, real-time applications, and clinical decision support systems. The Bidomain model [10,12,23,49,53] serves as the gold standard for describing the propagation of extra- and intracellular potentials in cardiac tissue, though its computational complexity presents significant challenges for large-scale simulations. In several works, in particular involving electromechanical coupling or interactions with fluid dynamics models of the human heart, researchers often turn to the more computationally efficient Monodomain model [12,23] as an alternative. The latter emerges as a simplified version of the Bidomain model where the intra- and extracellular conductivities are proportional. This simplification results in a model that maintains reasonable accuracy while significantly reducing the computational demand. The Monodomain model is constituted by a system of partial differential equations that describe the spatiotemporal evolution of the transmembrane potential and associated gating or recovery variables, which either represent the probability of ionic species flowing through the membrane or serve as recovery variables designed to reproduce observed phenomenological potentials (see, *e.g.*, the two-variable models derived from the FitzHugh-Nagumo model [19]). A more computationally efficient approach is provided by Eikonal models [12,23], which focus on the evolution of cellular excitation wavefronts rather than the complete spatial and temporal reconstruction of ionic action potentials: these models are extremely computationally cheap, though they provide less details regarding the propagation of the electrophysiological (EP) signals.

Despite the extensive range of mathematical models and numerical schemes available for addressing EP problems, their computational burden remains a significant concern. Furthermore, the primary interest in solving these models often lies in extracting key informative quantities that can significantly assist clinicians, such as activation and repolarization times within the cardiac domain. The activation and repolarization times serve as critical markers in cardiac electrophysiology, providing essential information about the heart’s electrical function: the activation time refers to the time when cardiac cells begin their depolarization process, whilst repolarization time denotes the time when cells return to their resting state. These markers are fundamental for understanding cardiac conduction patterns, identifying arrhythmogenic substrates [14], and evaluating the effects of drugs or interventions [3,56]. Traditional approaches for computing these times typically require solving the full Monodomain or Bidomain models, which can be computationally intensive depending on the geometry, and extracting the times from the resulting action potential waveforms. Moreover, while activation times can be evaluated more efficiently through the Eikonal model, repolarization times lack of a classical Eikonal-like counterpart.

The emergence of scientific machine learning offers new opportunities to address these computational challenges [9,20,25,32,39,42,51,52]. In particular, one of its main branches, operator learning, aims to approximate unknown operators that map between potentially infinite-dimensional functional spaces. Given pairs of input/output functional data (*u*,*f*), where u∈𝒰 and f∈𝒱 are functions defined on domains Ω and $\Omega^{\prime }$ respectively, the goal is to learn an approximation of an operator 𝒢:𝒰→𝒱 using machine learning architectures. Among the various recently-proposed operator learning architectures (see, *e.g*, [21,30,54]), Fourier Neural Operators (FNOs) [28] have emerged as a powerful approach based on the Neural Operator paradigm [26], parameterizing the integral kernel layers within the architecture in the Fourier space and allowing efficient learning of mappings between function spaces with resolution independence. FNOs have shown comparable performances for equispaced domains with respect to the vanilla Deep Operator Networks [31]. Another promising approach is Kernel Operator Learning (KOL) [5], which builds on standard kernel regression arguments to approximate the mapping between function spaces. Compared to other neural operator methodologies, the key advantage of KOL approach lies in its non-iterative formulation; the operator is obtained by solving a (potentially large) symmetric and positive definite linear system, thereby eliminating the need for iterative training procedures typically required in neural network-based frameworks.

**Fig 1:**
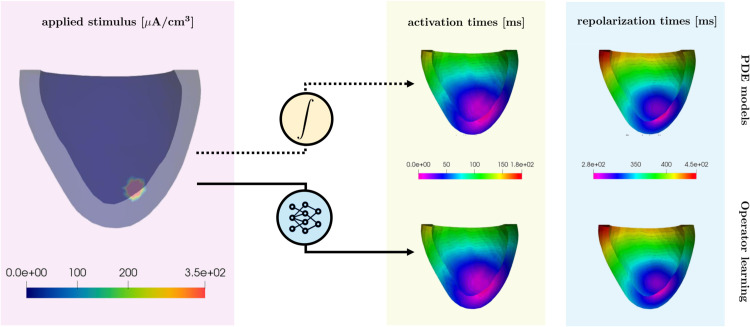
Schematic representation of the EP problem to address. In particular, we aim at reconstructing the activation/repolarization times of the cardiac tissue given the initial stimulus applied for 1 ms.

Using operator learning techniques to predict activation and repolarization times based on inputs such as tissue conductivity, fiber orientation, and stimulus location can help reduce computational bottlenecks in traditional cardiac modeling. These approaches will also improve efficiency, making computational tools more accessible for research and clinical practice. In this work, we take a step in this direction by learning the mapping between an initially applied current stimulus and the activation/repolarization times at each physical point in the considered 2D or 3D domains, as schematically represented in Fig 1. Specifically, we technically adapt FNO and KOL for the specific EP problem and compare the performances of FNO and KOL in terms of training time, test time, memory usage and accuracy in testing. In this way, we assess the potentialities of both strategies for retrieving fast and accurate simulations.

In summary, this study introduces novel applications of two promising operator learning schemes specifically tailored to cardiac electrophysiology problems and trained on *in-silico* data, with the potential to be extended to more realistic scenarios. A clinically relevant application of such surrogate models is in the context of inverse problems, such as reconstructing the site of origin of focal arrhythmias from observed activation maps. These inverse problems are typically solved by iteratively evaluating the forward model under different stimulation hypotheses, a process that is computationally expensive when using PDE-based models like the Monodomain or Bidomain formulations. Our operator learning approach aims to drastically accelerate this process by providing rapid approximations of activation/repolarization maps, thus enabling real-time or near real-time inference in future studies.

Furthermore, the proposed surrogate FNO and KOL models are also suited for training with real clinical data, even if it is out of the scope of this work. Indeed, activation times can be directly derived from extracellular potentials, which are routinely acquired in clinical practice: one possibility consists in computing the timing of the steepest negative slope (minimum variation of action potential) during the QRS complex [38,47,48]. Hence, endocardial or epicardial activation maps are routinely obtained in electroanatomical mapping procedures and can serve as meaningful input data for inverse modeling. Accordingly, the proposed setting provides significant value for clinical applications, particularly in identifying ectopic arrhythmia origins and guiding digitally assisted ablation procedures.

## Materials and methods

### PDE models

Mathematical models of electrophysiology play a crucial role in understanding and simulating the electrical activity of cardiac tissue. These models describe the evolution of the transmembrane potential and ionic currents, capturing the fundamental mechanisms of excitation and propagation.

There exists a wide variety of EP models for representing membrane dynamics and calcium handling at the cellular level, differing in complexity and physiological detail depending on the specific application (see, *e.g.*, [2,12,35,44]). On the other hand, spatial dynamics at the tissue scale are typically described by a few well-established macroscopic formulations, most notably the Monodomain and Bidomain [8,12] models. For more detailed modeling at the microscopic level, the Extracellular-Membrane-Intracellular (EMI) model [55] provides a cell-by-cell scale description.

The Monodomain model [57] is derived from the more challenging Bidomain model [1,6,8] when the intra- and the extracellular conductivity tensors, ${𝐃}_{i}$ and ${𝐃}_{e}$ respectively (measured in mS/cm), satisfy the following relationship [12]:

$${𝐃}_{e}=\lambda {𝐃}_{i}$$ (1)

where λ∈ℝ is a constant. The model reads as follows:

$$\left\{ \begin{array}{cc} \chi C_{m}\frac{\partial v}{\partial t}-\frac{\lambda }{1+\lambda }\text{div}({𝐃}_{i}\nabla v)+I_{\text{ion}}(v,𝐰)=I_{\text{app}} & \text{in }\Omega \times (0,T),\\ \frac{\partial 𝐰}{\partial t}-𝐑(v,𝐰)=0 & \text{in }\Omega \times (0,T),\\ \frac{\partial 𝐜}{\partial t}-𝐂(v,𝐰,𝐜)=0 & \text{in }\Omega \times (0,T),\\ {𝐧}^{⊤}{𝐃}_{i}\nabla v=0 & \text{on }\partial \Omega \times (0,T),\\ v(𝐱,0)=v_{0}(𝐱),\;𝐰(𝐱,0)={𝐰}_{0}(𝐱) & \text{in }\Omega . \end{array}\right.$$ (2)

where *v* is the transmembrane potential (in mV), which represents the difference between the intra- and extracellular potentials, *C*_*m*_ and χ are the membrane capacitance per unit area (in *μ*F/cm^2^) and the membrane surface area per unit volume (in cm^−1^) respectively, *λ* is the constant in (1), $I_{\text{ion}}$ is a ionic current density representing the flow of ionic species through the cellular membrane and $I_{\text{app}}$ is an applied stimulus in time, both measured in *μ*A/cm^3^. This latter term depends on *v*, as well as on the dimensionless gating or recovery variables of the ionic model, **w**. These variables either describe the probability of ionic species flowing through the membrane or serve as recovery variables designed to reproduce an observed phenomenological potential, as seen in two-variable models derived from the FitzHugh-Nagumo [22] model. They are coupled to the reaction diffusion PDE through a system of differential equations describing their evolution in time as well as the dynamics of the ionic concentrations **c** (in mM), which are ruled by often nonlinear functions 𝐑(v,𝐰) and 𝐂(v,𝐰,𝐜). If there is no injection of current in the extracellular space, (2) can be considered as a good approximation of the Bidomain model.

An alternative approach for modelling the evolution of the cellular excitation wavefront through PDE systems is based on Eikonal models [12,13,23,24]. In this case, the unknown is the activation time of cardiac cells, *i.e.* the instant when the transmembrane potential first crosses a predefined threshold during an action potential, marking the onset of electrical excitation. Eikonal models seem particularly attractive from a computational perspective compared to Monodomain, since they involve a single steady-state PDE that does not require coupling with ODE systems. More importantly, unlike the transmembrane potential, the activation time lacks internal or boundary layers, eliminating the need for special mesh restrictions. However, they require to solve a nonlinear PDE, which may be a bottleneck for real-time clinical applications or large-scale simulations.

Finally, we remark that activation and repolarization times can be both derived by post-processing the solution of the Monodomain model. However, while activation times can be obtained by solving Eikonal equations, no Eikonal-based formulation has been proposed in the literature to directly extract repolarization times.

In the next section, we introduce the operator learning tools that will enable us to construct surrogate models for both activation and repolarization times.

### Operator learning

Operator Learning (OL) aims to approximate an unknown operator


𝒢:𝒜→𝒰,

which maps functions from an input functional space (denoted by 𝒜) into the corresponding output functions belonging to another functional space (denoted by 𝒰). Given data pairs (*a*,*u*), where a∈𝒜 and u∈𝒰 are functions defined on bounded domains $\Omega \subset {\mathbb{R}}^{d}$, $\Omega^{\prime }\subset {\mathbb{R}}^{d^{\prime }}$ and sampled over ${\mathbb{R}}^{d}$ and ${\mathbb{R}}^{d^{\prime }}$ respectively, the goal is to learn an approximation of 𝒢 by means of machine learning surrogate model. The problem can be formalized as follows [26,28]:

**Problem 1.** *Let us consider $\{a_{i},u_{i}{\}}_{i=1}^{N}$ samples in 𝒜×𝒰, such that*

$$𝒢(a_{i})=u_{i},\;\text{with }i=1,2,\ldots ,N.$$ (3)

*We define the observation operators $\phi :𝒜\to {\mathbb{R}}^{d_{a}}$ and $\varphi :𝒰\to {\mathbb{R}}^{d_{u}}$ acting on the input and the output functions, respectively. These operators represent discretizations of the underlying function, which are commonly used in practical applications. The aim of operator learning is approximating the operator 𝒢 through the observation of input/output pairs $\left\{\phi (a_{i}),\varphi (u_{i})\right\}$.*

In this work we consider a natural choice for the observation operators *ϕ* and φ, namely the pointwise evaluation at specific collocation points in Ω and $\Omega^{\prime }$, respectively. In particular, since in our setting $\Omega \equiv \Omega^{\prime }$, we will also take the same collocation points for both *ϕ* and φ.

Furthermore, we work within a supervised learning framework, where, among all the possible operators ranging between 𝒜 and 𝒰, we aim at finding the one which minimizes the error on the observed (training) couples assuming tailored paradigms for the approximated operator, *e.g.* machine learning architectures. In the following, we briefly expand on the two methods employed for the reconstruction of activation and repolarization times, namely Fourier Neural Operators and Kernel Operator Learning.

### Fourier neural operators

**Fig 2:**
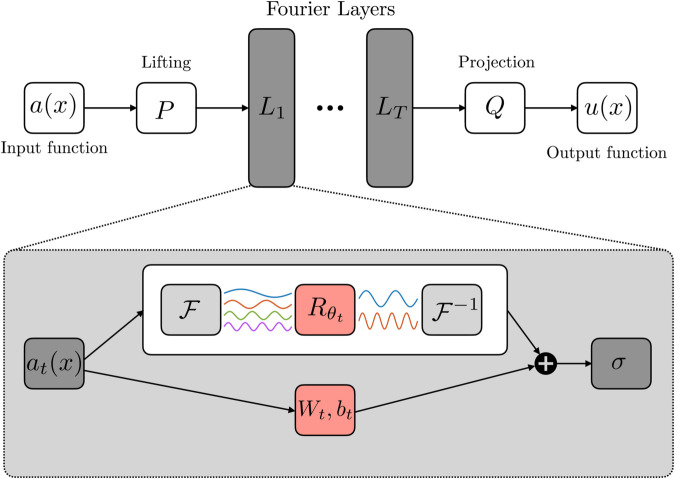
Schematic architecture of Fourier Neural Operators (FNO).

Fourier Neural Operators (FNOs) [28] belong to the broader class of operator learning frameworks known as Neural Operators (NOs) [26]. The NO paradigm defines a sequence of functions $a_{0}\mapsto a_{1}\mapsto \ldots \mapsto a_{T}$, with each *a*_*t*_ having values in ${\mathbb{R}}^{d_{a_{t}}}$ for t=0,1,…,T − 1. As shown in Fig 2, the input a∈𝒜 is first lifted pointwise by a transformation *P*, resulting in *a*_0_(*x*) = *P*(*a*(*x*)). The function is then propagated through a series of updates ($a_{t}\mapsto a_{t+1}$), and the final output is projected as *u*(*x*) = *Q*(*a*_*T*_(*x*)) in order to belong to the same space of the function to reconstruct, where $Q:{\mathbb{R}}^{d_{a_{T}}}\to {\mathbb{R}}^{d_{u}}$ is another pointwise transformation. Each update step combines a non-local integral operator with a nonlinear activation, as follows:

$$a_{t+1}(x)=\sigma_{t+1}\left(W_{t}a_{t}\left(x\right)+\int_{\Omega_{t}}\kappa^{\left(t\right)}(x,y)a_{t}(y)\;d\nu_{t}(y)+b_{t}(x)\right)\;\forall x\in {\mathbb{R}}^{d_{a_{t+1}}}.$$ (4)

FNOs arise by restricting the kernel to be translation-invariant, i.e., $\kappa^{\left(t\right)}(x,y)=\kappa^{\left(t\right)}(x-y)$, and leveraging the convolution theorem in the Fourier domain. Denoting by ℱ the Fourier transform and by ${ℱ}^{-1}$ its inverse, the integral operator is thus computed as:

$$(K_{t}(a_{t}))(x)={ℱ}^{-1}\left(R_{\theta_{t}}\cdot ℱ(a_{t})\right)(x),\;\forall x\in \Omega_{t}\subset {\mathbb{R}}^{d_{a_{t}}},$$ (5)

where $R_{\theta_{t}}$ denotes the truncated, learnable Fourier coefficients of the kernel. When the domain is discretized through evenly spaced points, Fast Fourier Transform (FFT) can be employed for computing efficiently (5). For further technical details, we refer the interested reader to Text A in S1 File.

### Kernel operator learning

**Fig 3:**
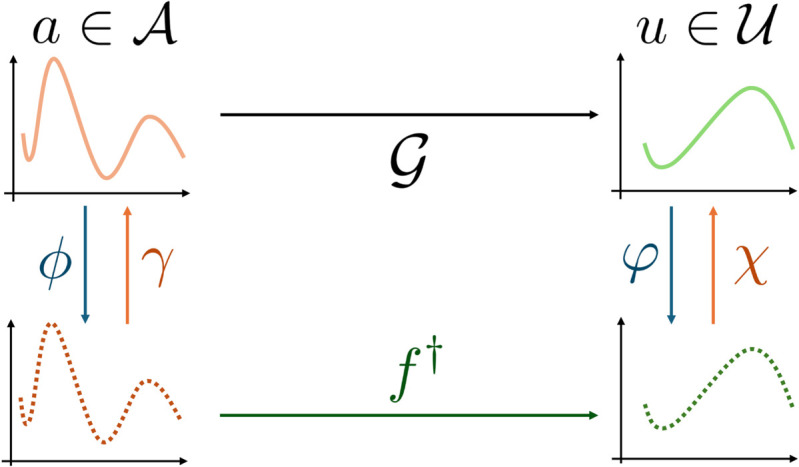
Kernel Operator Learning (KOL) diagram. Starting from the input function *a*, *A* collects observations of the function at different collocation points through *ϕ*. Then, the vector-valued *f*^†^ processes observations of the input into observations of the output A=ϕ(a). Finally, the reconstruction operator is applied to determine the output function u=χ(U).

Kernel Operator Learning (KOL) is a recently formalized operator learning technique [5], based on standard kernel regression arguments. Following the diagram in Fig 3, retrieving the approximated operator $\bar{𝒢}$ is equivalent to determining a vector-valued function $f^{†}:{\mathbb{R}}^{n\cdot d_{a}}\to {\mathbb{R}}^{n\cdot d_{u}}$ ranging between discrete observations of the input to observations of the output. Endowing 𝒜, 𝒰 and the space in which we look for *f*^†^ with Reproducing Kernel Hilbert Space (RKHS) structures, the approximated operator can be written explicitly in closed form as

$$\bar{𝒢}(a)(𝐱)=K(𝐱,𝒳)K(𝒳,𝒳{)}^{-1}\left(\sum_{j=1}^{N}S(\phi (a),A_{j})\alpha_{j}\right),$$ (6)

where *K* is the kernel function induced by the RKHS structure of 𝒰, the vector


$$𝒳=[{𝐱}_{1}^{T},{𝐱}_{2}^{T}\ldots {𝐱}_{n}^{T}{]}^{T}\in {\mathbb{R}}^{n\cdot d}$$

contains the collocation points, and $S:{\mathbb{R}}^{n}$ × ${\mathbb{R}}^{n}\to \mathbb{R}$ is a properly chosen vector-valued kernel generating the space of *f*^†^ functions. Moreover, $K(\cdot ,𝒳):\Omega \to {\mathbb{R}}^{n}$ is a row vector such that $K(𝐱,𝒳{)}_{i}=K(𝐱,{𝐱}_{i})$, and K(𝒳,𝒳) is an *n* × *n* matrix such that $K(𝒳,𝒳{)}_{ij}=K({𝐱}_{i},{𝐱}_{j})$. Since we deal with pointwise observation functions, K(x,𝒳) computes the evaluation of the linear interpolant of points in 𝒳 at **x**, whilst $K({𝐱}_{i},{𝐱}_{j})=\delta_{i}^{j}$. Parameters $\{\alpha_{j}{\}}_{j=1}^{N}\in {\mathbb{R}}^{n}$ are the kernel regression parameters over the input/output training pairs. We refer to [60] for the mathematical derivation of the explicit representation of KOL operator and more details.

From a computational standpoint, after selecting the scalar kernel S(·,·) for the discrete vector spaces, the problem reduces to solving *n* linear systems of size *N* in order to determine the different components of each $\{\alpha_{j}{\}}_{j}$. To achieve this, we employ the Cholesky factorization of the matrix and solve the resulting systems using standard substitution methods. Moreover, a regularization term is introduced in the regression formulation, with a penalty parameter set to 10^−10^.

A key factor influencing the approximation and generalization properties of KOL methods is the selection of the scalar kernel *S*. The optimal kernel function for kernel regression remains a subject of ongoing debate and is application-dependent, as it directly influences the accuracy of the trained architecture. Consequently, performing sensitivity analyses is essential to identify the kernel that maximizes accuracy for the specific application. For example, some novel approaches involve learning kernels by simulating data-driven dynamical systems, enhancing the scalability of Kernel Regression [36]. In general, any symmetric positive semi-definite function that maps two elements of the vector space to a positive value can serve as a kernel. In this study, we consider few choices for *S*: Radial Basis Functions (RBF), Neural Tangent Kernel (NTK) and the kernel generated by the Euclidean distance between centroids of the initially activated spots (IQ). For the mathematical definitions of those functions we refer to Text B in S1 File. We note that the latter proposed kernel is specifically tailored for input functions that represent cardiac stimuli: it is often necessary to appropriately adjust the kernel functions based on the specific application at hand. This kernel is particularly effective in reconstructing activation and repolarization maps, whose iso-contours vary consistently with the distance from the activation site.

## Results and discussion

In this section we report the results of the numerical tests performed with FNO and KOL on three different test cases for our problem: a 2D squared domain (Subsection 2D case), a 3D slab (Subsection 3D slab) and a realistic ventricle unstructured mesh (Subsection 3D unstructured ventricle).

### Dataset generation and computational details

**Fig 4:**
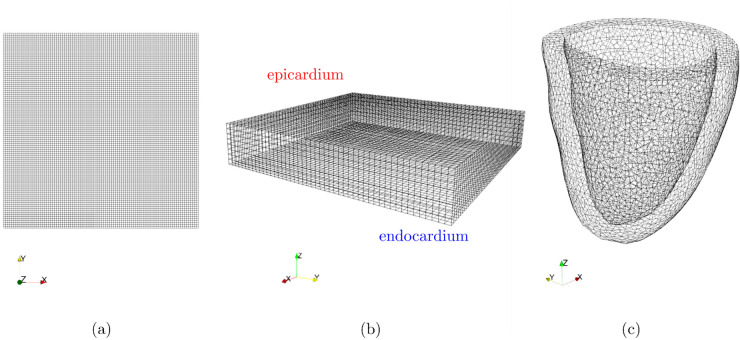
Grids adopted for the numerical simulations: (a) 2D grid (structured, 100×100 elements, physical area 1 cm × 1 cm = 1 cm^2^), (b) 3D slab (structured, 384×384×64 elements, physical volume 3.84 cm × 3.84 cm × 0.64 cm = 9.44 cm^3^. The figure displays the coarser 48×48×8 elements grid used to save the dataset employed to train the operator learning models.) and (c) 3D unstructured ventricle (about 35k nodes, physical volume 100 cm^3^). For all the geometries, we have considered Q1 elements (regular squares/cubes for structured 2D and 3D cases, hexaedral elements for the 3D unstructured case).

To construct the dataset for training operator learning models, we generate input excitations and the corresponding solutions of the Monodomain model (2) discretized by Q1 finite elements, on quadrilateral grids for the 2D case and hexahedral grids for the 3D case (see Fig 4). The Monodomain model is coupled with either the Rogers-McCulloch ionic model [43] for the 2D case or the Ten Tusscher ionic model [50] for the 3D cases, in order to describe the transmembrane potential between the intra- and extracellular domains. Activation and repolarization times are computed as postprocessing of the Monodomain solutions. The applied excitation is defined as a fixed intensity pulse applied for 1ms over a random location of the domain. Regarding the diffusion parameters, we have considered $C_{m}=1\mu$F/cm^2^, χ=1cm^−1^, and ${𝐃}_{i}=\sigma_{1}{𝐧}_{1}{𝐧}_{1}^{T}+\sigma_{2}{𝐧}_{2}{𝐧}_{2}^{T}$  +  $\sigma_{3}{𝐧}_{3}{𝐧}_{3}^{T}$, where the triplet $\left\{{𝐧}_{1},{𝐧}_{2},{𝐧}_{3}\right\}$ represents the fiber orientation. The conductivities employed in the various scenarios are chosen according to the test cases outlined in [12].

All training and test samples exhibited conduction velocities (CVs) that align with the established physiological range reported in experimental and computational studies. Specifically, in the 2D and the 3D slab case we measure 0.06 cm/ms along-fiber CV and 0.03 cm/ms across, whilst in the 3D unstructured case we computed a CV of 0.05 cm/ms along-fiber CV and 0.02 cm/ms across using both a simulation on a cable model with the same conductivity parameters and the formula $\nabla T/|\nabla T{|}^{2}$, where *T* is the activation time expressed as a function of the position on the cardiac tissue [16]. The slightly slower propagation observed in the latter can be attributed to the coarser mesh resolution, which may lead to longer activation times as visible in Figs 18 and 19. Nonetheless, these values remain consistent with the physiological range reported for mammalian cardiac tissue in [11,15].

For each training we consider an input dataset collecting electrical stimuli (named iapps) and the corresponding output maps of activation times (named acti) and repolarization times (named repo). The name of each dataset is followed by the number of samples it contains, where the repartition between training and test dataset is 80%/20%. The datasets’ structure varies depending on the spatial dimension, as detailed below:

- **2D case**: we consider *N* different current stimuli in iapps in the form of N×2 matrices, collecting the (*x*,*y*) coordinates where each pulse is applied. For the basic case, activation and repolarization times are two matrices named of size $N\times n_{\text{no}}^{2}$, where $n_{\text{no}}$ is the number of discretization points per dimension (101 in our case), and ${𝐧}_{1}={𝐧}_{x}$ and ${𝐧}_{2}={𝐧}_{y}$. In this scenario, we have also considered the case where the cardiac fibers are rotated by 45^°^ counterclockwise around the *z* axis, with the rotation applied outward from the *xy* plane, and the corresponding output maps of activation and repolarization times are called acti rot and repo rot, respectively. The sizes of these datasets are the same of the basic case.
- **3D slab**: each stimulus is an $N\times n_{x}\times n_{y}\times n_{z}$ binary tensor, where ones indicate pulse application points. We consider $n_{x}=n_{y}=49$ nodes in the *x* and *y* directions, *n*_*z*_ = 9 nodes in the *z* direction, extracted through a projection from a larger 385×385×65 mesh. The activation and repolarization outputs have the same shape of the pulse. For the fiber direction we assume ${𝐧}_{1}={𝐧}_{x},\;{𝐧}_{2}={𝐧}_{y}$ and ${𝐧}_{3}={𝐧}_{z}$.
- **3D unstructured ventricle**: the input stimuli are $N\times N_{n}\times 3$ matrices, where *N*_*n*_ is the number of nonzero nodes in the unstructured mesh of the ventricle. Similarly, activation and repolarization outputs are $N\times N_{u}\times 3$ tensors, with *N*_*u*_ being the total number of unstructured mesh nodes (about 35k DOFs, 30k elements extracted from a finer 1.98 mln DOFs mesh with 1.93 mln elements – in order to deal with activation and repolarization times lying in realistic human pathological ranges [7,17]). In this case we consider the fiber orientation extracted by the physiological ventricle following the procedure described in [37]. This geometry is characterized by the following physical dimensions: maximum height of 7.23 cm, Left Ventricular End-Diastolic Diameter (LVEDD) of 5.85 cm, and Interventricular Septal Thickness in Diastole (IVSd) of 1.08 cm.

The generation of a single sample required approximately 8 minutes for the 2D case on an Intel Core i5 quad-core (2.7 GHz) processor, and 15–20 minutes for the 3D cases on a machine equipped with an NVIDIA Quadro RTX 5000 GPU. In Table 1 we report further details about the parameters employed for each case.

**Table 1:** Parameters of the high-fidelity solvers used for the 2D, 3D, and 3D unstructured cases. Here, *h* is the element diameter, *dt* the time step, $T_{\text{sti}}$ the stimulation duration, $\chi C_{m}$ the membrane capacitance per unit surface area, $\sigma_{1}$, $\sigma_{2}$, $\sigma_{3}$ the conductivities along the principal directions, and $I_{\text{ap}}$ the amplitude of the applied stimulus current density and $T_{\text{sim}}$ is the simulated physical time. The 2D case has been solved using MATLAB’s direct solver, while the 3D cases have been solved using the Conjugate Gradient (CG) solver provided by the PETSc library and Hypre BoomerAMG as preconditioner [4,59].

Case of study	2D	3D	3D unst.
DOFs	101×101	385×385×65 (proj. 49×49×9)	1.98 mln (proj. 35k)
*h* [cm] (diameter)	0.01	0.01 (proj. 0.08)	0.05 (avg.) (proj. 0.18 (avg.))
*dt* [ms]	0.1	0.05	0.05
$T_{\text{sti}}$ [ms]	1	1	1
$T_{\text{sim}}$ [ms]	150	500	500
$\chi C_{m}$ ([*μ*F/cm^2^] or [*μ*F/cm^3^])	1	1	1
$\sigma_{1}$ [mS/cm]	1.2E-03	1.2E-03	1.2E-03
$\sigma_{2}$ [mS/cm]	2.6E-04	2.6E-04	2.6E-04
$\sigma_{3}$ [mS/cm]	–	2.6E-04	2.6E-04
$I_{\text{ap}}$ ([*μ*A/cm^2^] or [*μ*A/cm^3^])	200	350	250
Ionic Model	RM [43]	TT06 [50]	TT06 [50]
Solver	MATLAB backslash	CG+Hypre (PETSc)	CG+Hypre (PETSc)

These structured and unstructured datasets provide diverse inputs for training operator learning architectures. In order to evaluate accuracy performance of the trained OL schemes we compute the generalization error as the discrete *L*^2^ relative error, namely,

$$L_{\text{err}}^{2}=\frac{1}{N_{p}}\sum_{n=1}^{N_{p}}\frac{\|y_{p}^{n}-y^{n}{\|}_{2}}{\|y^{n}{\|}_{2}},$$ (7)

where $y^{n}$ is the vector containing the ground truth evaluations of activation/repolarization maps at the different points of the domain whilst $y_{p}^{n}$ is the corresponding vector of predictions. For the 3D case representing a 3D ventricle we also compute the Pearson dissimilarity coefficient *P* is defined as *P* = 1−*R*, with

$$R=\frac{\text{Cov}(Y,Y_{p})}{\sigma_{Y}\sigma_{Y_{p}}}.$$ (8)

Here, $\text{Cov}(Y,Y_{p})$ is the covariance between the ground truth of the tested samples and the predictions as flattened vectors (respectively *Y* and *Y*_*p*_) and $\sigma_{Y,Y_{p}}$ is the standard deviation relative to the test dataset or the predictions. This index is commonly employed in machine learning to quantify the correlation between target outputs and their reconstructed counterparts.

Finally, we remark that in both 2D and 3D cases FNO architectures were trained on a workstation with an NVIDIA Quadro RTX 5000 GPU, while KOL was trained on an Intel i7 CPU. For a fair comparison from the end-user perspective, all inference timing analyses were conducted on a single device, a standard laptop equipped with an Apple M1 Pro chip (CPU only).

### 2D case

**Fig 5:**
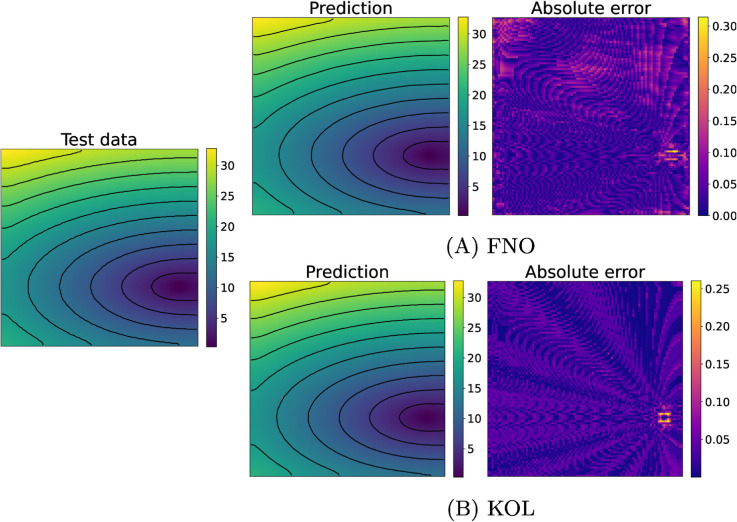
Comparison of FNO (A) and KOL (B) activation time predictions for the 2D case (acti with 2000 samples). Colorbars indicate time in milliseconds (ms).

**Fig 6:**
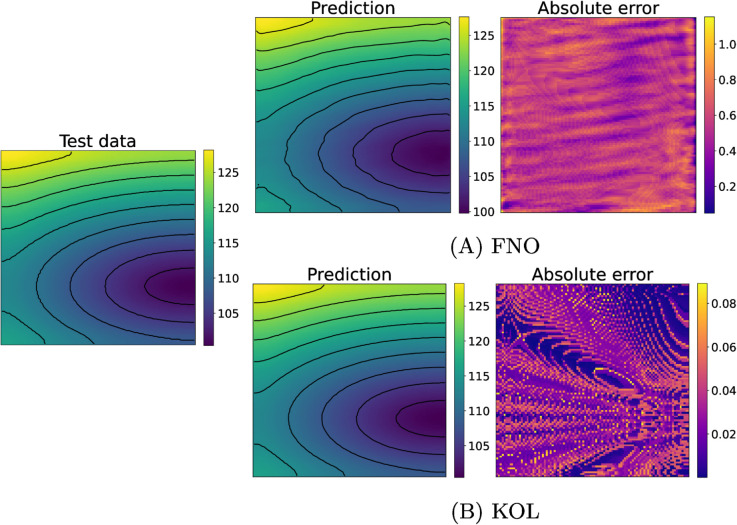
Comparison of FNO (A) and KOL (B) repolarization time predictions for the 2D case (repo with 2000 samples). Colorbars indicate time in milliseconds (ms).

Figs 5 and 6 depict test samples reconstructed with FNO (top) and KOL (bottom) for activation and repolarization times. The FNO architecture employed comprises four Fourier layers with 16 Fourier modes along the *x*-axis and 4 modes along the *y*-axis, with a total number of approximately 532k trainable parameters. In Tables 2 and A in S1 File, results indicate that the use of a tailored learning rate reduction policy (reduce- OnPlateau), where the learning rate is reduced by a factor of 0.95 whenever the test loss is not decreasing, consistently outperforms training without such a policy. For instance, in the activation dataset acti with 3000 samples, the test error is reduced from 2.82 × 10^−3^ (no policy) to $2.66\times 10^{-3}$ when the policy is applied. In the FNO results the uncertainty bands arise from considering different trained architectures with various Kaiming normal initializations [18] of the trainable parameters.

**Table 2:** Performance comparison of FNO (reduce-on-plateau learning rate policy) and KOL (iq4 kernel) methods on 2D datasets.

	Dataset (size)	Test Error	GPU Memory	Training Time	Test Time
FNO with lr policy reduceOnPlateau	acti (3000)	2.66E-03 ± 2.27E-04	5.79GB	72 min	5.7E-05 sec
	acti rot (2000)	3.33E-03 ± 3.13E-04	5.62GB	48 min	5.9E-05 sec
	repo (3000)	3.13E-03 ± 2.33E-04	5.79GB	72 min	4.0E-05 sec
	repo rot (2000)	3.53E-03 ± 3.63E-04	5.62GB	48 min	5.4E-05 sec
	**Dataset (size)**	**Test Error**	**CPU Memory**	**Training Time**	**Test Time**
KOL with iq4 kernel	acti (3000)	9.52E-04	1.15GB	10 min	6.3E-04 sec
	acti rot (2000)	9.34E-04	0.91GB	8 min	2.2E-04 sec
	repo (3000)	4.74E-04	1.15GB	10 min	7.1E-04 sec
	repo rot (2000)	4.69E-04	0.91GB	6 min	2.9E-04 sec

The rotated fiber tests, where a 45^°^ rotation of the myocytes is applied on the domain, generally show larger test errors compared to their non-rotated counterparts with the same number of training samples (cfr. Tables A and B in S1 File). This observation suggests that the FNO architecture might be sensitive to the structural alignment of input features. Moreover, we notice that increasing the dataset size improves the performance, as evident in both activation and repolarization datasets. For activation tests with 2000 and 3000 samples, the test error drops, highlighting the benefit of having more training data for learning the spatial features of the solution. In terms of computational efficiency, for FNO the GPU memory consumption remained stable at approximately 5.6GB for 2000-sample datasets and increased slightly to 5.79GB for 3000-sample datasets. Training times scaled proportionally with dataset size, from 48 minutes for 2000 samples to 72 minutes for 3000 samples. We notice a general low absolute error, with some areas for the activation case propagating orthogonally with respect to level curves. This error distribution was observed also for the KOL case in Fig 5 (bottom, right), but a theoretical explanation of such a pattern remains unknown. A more uniform pattern is observed instead for the repolarization case.

We conducted a large number of numerical simulations using the same training and test datasets employed for FNO architectures to evaluate the performance of KOL (cfr. Tables 2, B and C in S1 File). The primary objective of this sensitivity analysis was to assess the impact of kernel selection, a critical factor that is highly problem-dependent and can significantly affect prediction quality.

From Tables B and C in S1 File, we observe that IQ kernel-based strategies yield generalization errors at least two orders of magnitude lower than those using NTK or RBF. This improved performance is attributed to the IQ kernel’s efficiency in computing correlations of compact support indicator functions, which accurately represent activation regions. Notably, this advantage persists even when increasing the training set size for both activation and repolarization reconstructions. Additionally, KOL exhibits sensitivity to structural alignments of input features. Specifically, tests with rotated fibers achieve higher accuracy than unrotated counterparts. We observe that KOL significantly reduces training times with respect to FNO, decreasing from thousands of seconds to just few hundreds, as it requires solving a single symmetric, positive definite linear system rather than an iterative optimization process. However, as training size increases, the system’s condition number rises which may impact on testing performances. Therefore, in this case, the use of tailored preconditioning strategies is crucial (cfr. [33,46]). Regarding computational efficiency, CPU memory consumption scales with training size but remains close to 1 GB. It is important to note that the accuracy and training time improvements offered by KOL must be considered in light of the non-negligible time required for kernel selection. Furthermore, we note that KOL, equipped with the chosen deterministic kernels, produces fully deterministic predictions: therefore, unlike FNO, it does not exhibit uncertainty bands due to hyperparameter initialization.

### 3D slab

**Fig 7:**
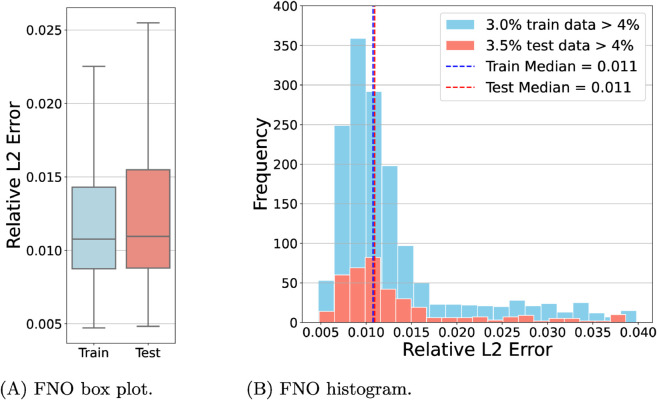
FNO box plot (A) and histogram (B) for the 3D dataset acti 2000 relative to the best model trained. For the training set, 3% of the data have a relative L2 error greater than 4%, while for the test set, 3.5% of the data exceed this threshold.

**Fig 8:**
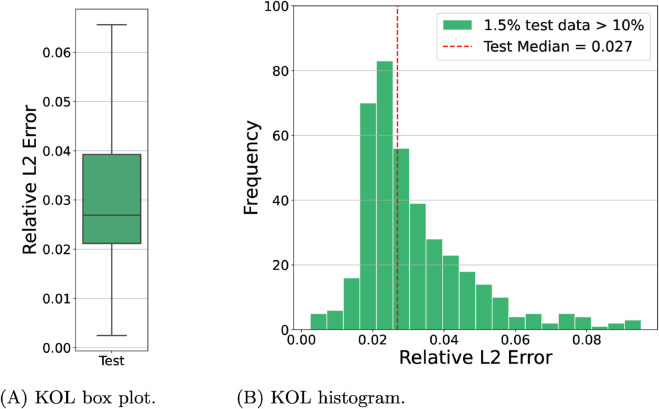
KOL box plot (A) and histogram (B) for 3D dataset acti 2000 relative to the best model trained. Training results are not shown since we achieve machine precision. For the test set, 1.5% of the data have a relative L2 error greater than 10%.

**Fig 9:**
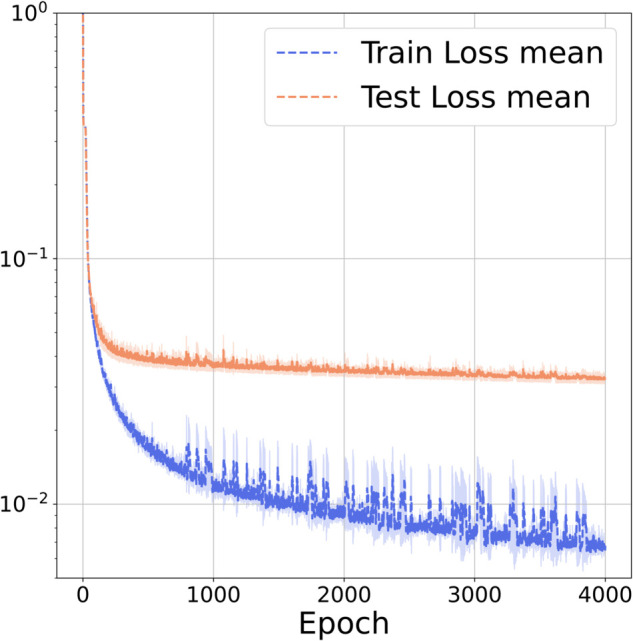
FNO loss plot for the 3D case (acti 2000) dataset). Mean train and test loss of three different randomly initialized models (dashed line). Standard deviation over the three models for each epoch is also reported (light shadow).

Additional experiments on a 3D slab in (0,1)^3^ have been conducted using the two operator learning approaches discussed. As will be shown, the performance of FNO and KOL remained robust despite the increased dimensionality of this test case. The architecture employed for FNO comprises four Fourier layers with 16 Fourier modes along the *x*-axis, 8 along the *y*-axis and 4 along the *z*-axis, resulting in approximately 8.4 million trainable parameters. Given the improved performance observed for the 2D case, all experiments used the reduceOnPlateau learning rate policy, where the learning rate decreases upon stagnation of the validation loss. Instead, we endow KOL with iq4 kernel (cfr. Table E in S1 File) following the sensitivity analysis of the 2D case. In Table 3, the results indicate that increasing the dataset size significantly enhances prediction accuracy for both models. For the activation dataset, the FNO test error decreased from 4.15 × 10^−2^ to 3.27 × 10^−2^ when the sample size increased from 1000 to 2000. A similar trend is observed for repolarization, where the test error dropped from 8.46 × 10^−3^ to 6.91 × 10^−3^. Conversely, KOL exhibits the opposite behavior: as the dataset size grows, the conditioning number of the SPD system increases, and, therefore test error increases (*e.g.* from 5.33 × 10^−3^ at 1000 dataset size to 6.19 × 10^−3^ at 2000 dataset size for repolarization). Nonetheless, test errors for KOL remain consistently lower than those of FNO in both activation and repolarization cases. However, as reported in Fig 7, most of the test data predictions for the acti 2000 dataset are distributed under 1% of the relative L2 error, despite having around 3.5% of the data as outliers for the FNO case. Similar results are obtained for KOL in Fig 8. For the repo 2000 dataset the reader can refer to Figs A and B in S1 File. In Fig 9 we have also reported the training and test loss decaying plot for FNO.

**Table 3:** Performance comparison of FNO and KOL methods on 3D datasets.

	Dataset (size)	Test Error	GPU Memory	Training Time	Test Time
FNO with lr policy reduceOnPlateau	acti (1000)	4.15E-02 ± 2.02E-03	13.66GB	94 min	4.6E-04 sec
	acti (2000)	3.27E-02 ± 5.86E-04	14.09GB	188 min	2.4E-04 sec
	acti hetero (2000)	2.75E-02 ± 6.31E-04	14.09GB	188 min	2.6E-04 sec
	repo (1000)	8.46E-03 ± 6.51E-04	13.66GB	94 min	4.8E-04 sec
	repo (2000)	6.91E-03 ± 3.44E-04	14.09GB	188 min	2.4E-04 sec
	repo hetero (2000)	6.00E-03 ± 8.76E-04	14.09GB	188 min	3.0E-04 sec
	**Dataset (size)**	**Test Error**	**CPU Memory**	**Training Time**	**Test Time**
KOL with iq4 kernel	acti (1000)	1.67E-02	1.67GB	3 min	1.0E-03 sec
	acti (2000)	1.82E-02	2.56GB	7 min	1.4E-03 sec
	acti hetero (2000)	1.36E-02	2.56GB	12 min	1.4E-03 sec
	repo (1000)	5.33E-03	1.67GB	3 min	1.2E-03 sec
	repo (2000)	6.19E-03	2.56GB	7 min	2.0E-03 sec
	repo hetero (2000)	2.59E-03	2.56GB	12 min	2.0E-03 sec

**Fig 10:**
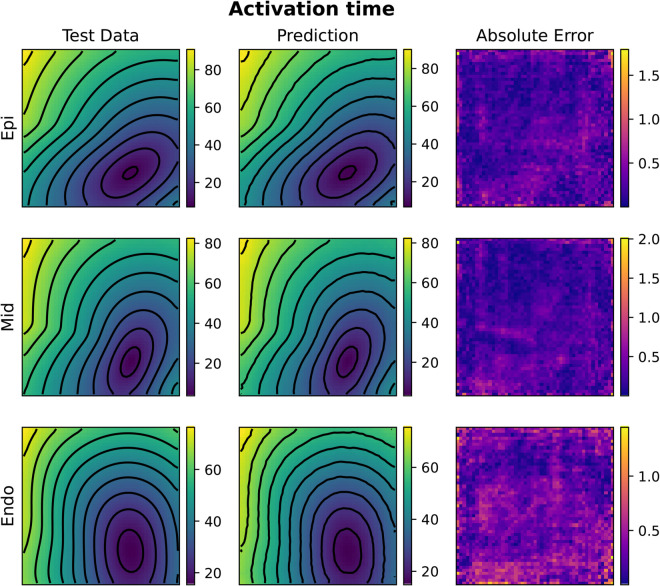
Example of FNO activation time prediction for the 3D case (acti with 2000 samples). The picture represents three slices of tissue: epicardium (top), middle (center) and endocardium (bottom). Colorbars indicate time in milliseconds (ms).

**Fig 11:**
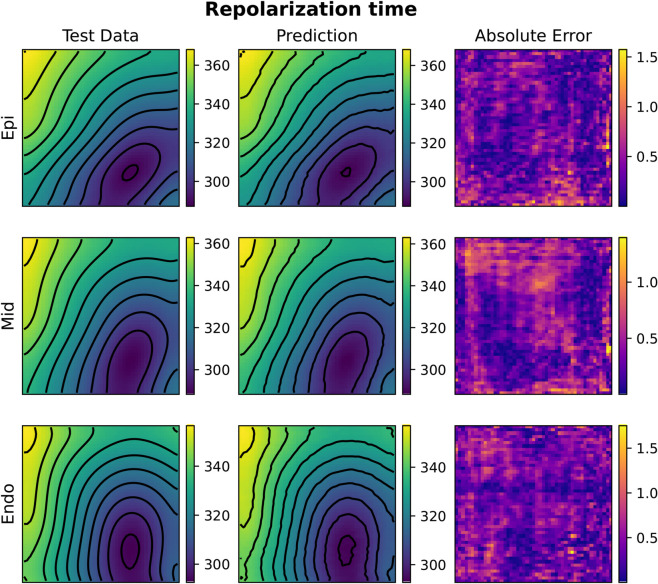
Example of FNO repolarization time prediction for the 3D case (repo with 2000 samples). The picture represents three slices of tissue: epicardium (top), middle (center) and endocardium (bottom). Colorbars indicate time in milliseconds (ms).

**Fig 12:**
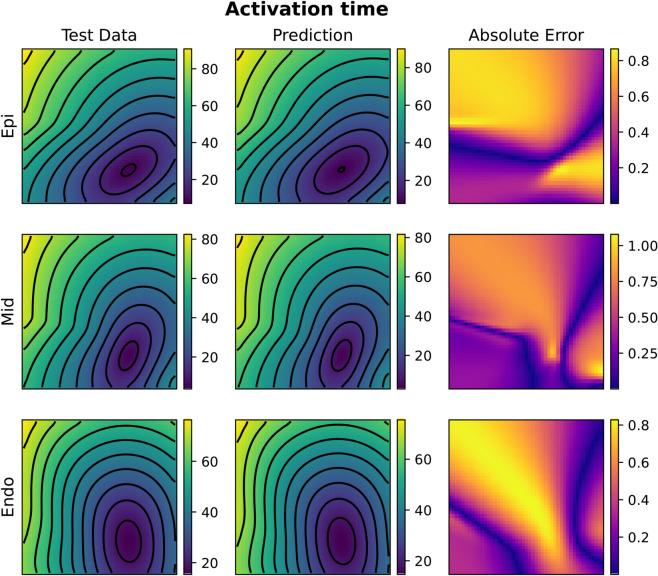
Example of KOL activation time prediction for the 3D case (acti with 2000 samples). The picture represents three slices of tissue: epicardium (top), middle (center) and endocardium (bottom). Colorbars indicate time in milliseconds (ms).

**Fig 13:**
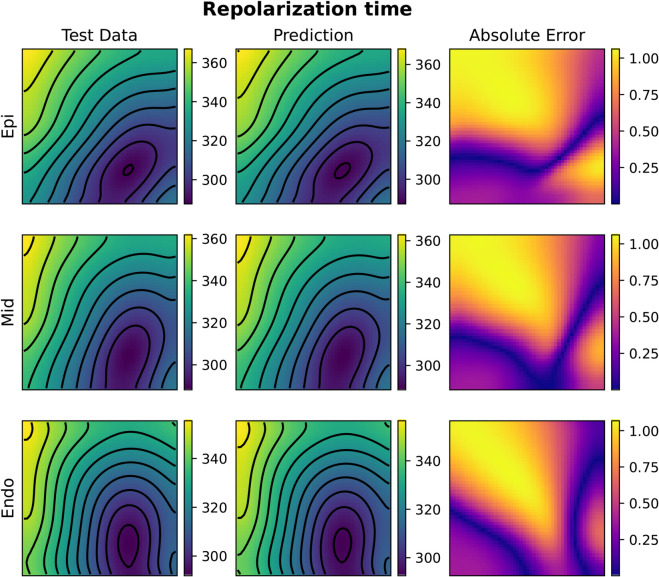
Example of KOL repolarization time prediction for the 3D case (repo with 2000 samples). The picture represents three slices of tissue: epicardium (top), middle (center) and endocardium (bottom). Colorbars indicate time in milliseconds (ms).

Compared to 2D tests, computational costs rise substantially in 3D. For FNO, GPU memory consumption increased from 13.66GB (1000 samples) to 14.09GB (2000 samples), whereas KOL required 1.67GB and 2.56GB, respectively. Training times scaled linearly for both models, with FNO requiring 94 minutes for 1000 samples and 188 minutes for 2000, while KOL completed training in 211 seconds and 427 seconds for the same dataset sizes. Figs 10, 11, 12 and 13 illustrate activation and repolarization time predictions for both models, along with the corresponding high fidelity solutions and absolute errors across three slices of the slab domain representing the endocardium, epicardium and the intermediate slice. The applied stimulus belongs to the epicardium surface for the reconstruction of activation times, whilst it belongs to an intermediate sheet between the endocardium and the middle of the slab for the repolarization case. In both cases, prediction error increases as far as the distance from applied stimuli increase. While FNO exhibits a nearly uniform absolute error distribution slightly higher than in the 2D case, KOL’s errors tend to be concentrated near the activation region and propagate orthogonally to the level curves of the activation (or repolarization) times.

Additionally, we investigate on spatial and cellular heterogeneity across the myocardium, such as differences between endocardial, epicardial, and midmyocardial cells, which represents a significant challenge in electrophysiological modeling. The following results indicate that the proposed learning framework is able to handle key forms of electrophysiological heterogeneity and can be extended to more complex tissue-specific scenarios.

**Fig 14:**
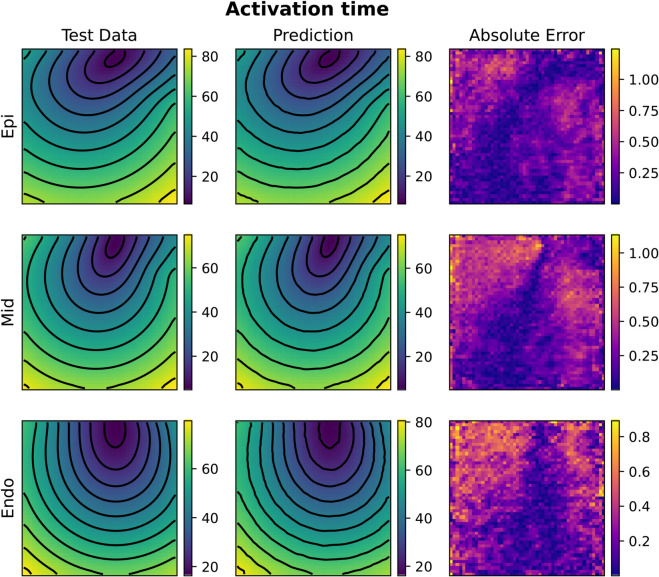
Example of FNO activation time prediction for the 3D heterogeneous case (acti with 2000 samples). The picture represents three slices of tissue: epicardium (top), middle (center) and endocardium (bottom). Colorbars indicate time in milliseconds (ms).

**Fig 15:**
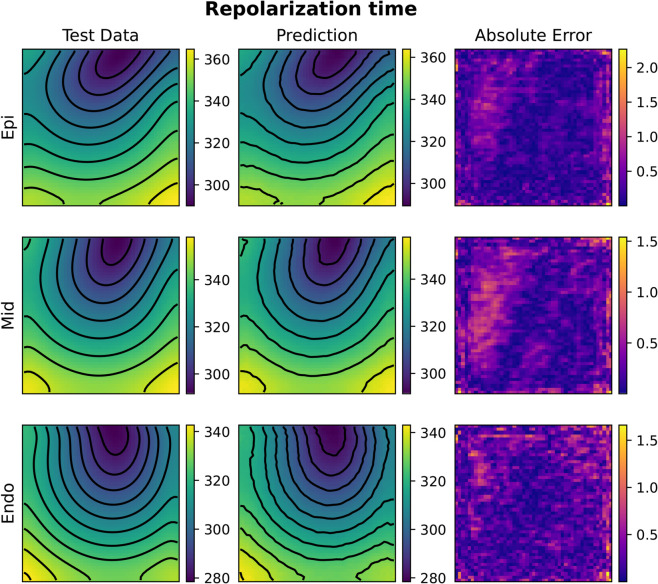
Example of FNO repolarization time prediction for the 3D heterogeneous case (repo with 2000 samples). The picture represents three slices of tissue: epicardium (top), middle (center) and endocardium (bottom). Colorbars indicate time in milliseconds (ms).

**Fig 16:**
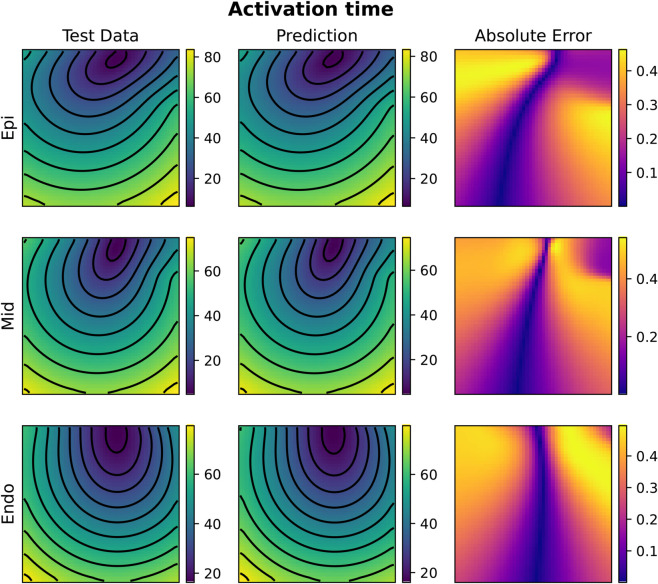
Example of KOL activation time prediction for the 3D heterogeneous case (acti with 2000 samples). The picture represents three slices of tissue: epicardium (top), middle (center) and endocardium (bottom). Colorbars indicate time in milliseconds (ms).

**Fig 17:**
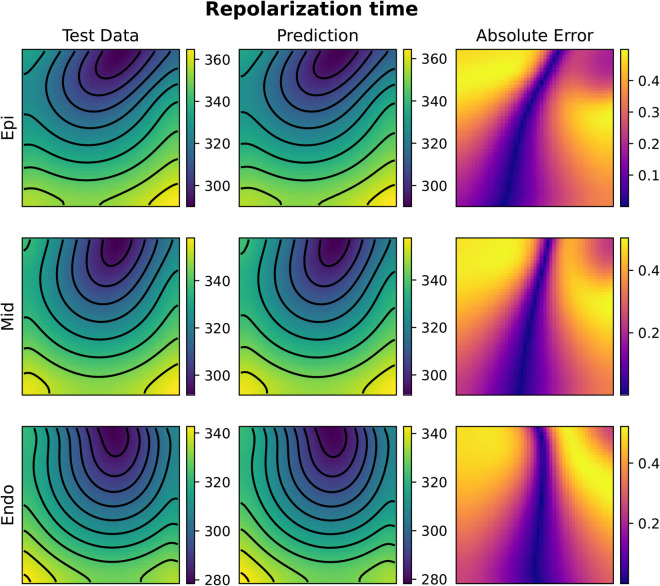
Example of KOL repolarization time prediction for the 3D heterogeneous case (repo with 2000 samples). The picture represents three slices of tissue: epicardium (top), middle (center) and endocardium (bottom). Colorbars indicate time in milliseconds (ms).

We have conducted experiments in the 3D case incorporating spatial heterogeneity in action potential duration (APD) by varying the *I*_*Ks*_ conductance in the ten Tusscher ionic model [50]. The conductance parameter varies across nine myocardial layers to reflect physiological differences in cardiac tissue: the bottom three layers maintain the base conductance, the middle three layers exhibit a reduced conductance (70% of the base value), and the top three layers show an increased conductance (140% of the base value). This setup allows us to assess the robustness of the learning framework in the presence of realistic electrophysiological variability. As reported in Table 3, both the Fourier Neural Operator and Kernel Operator Learning models retained the expected test accuracy in this heterogeneous setting. In particular, the FNO achieved a relative test error of 2.69% for activation and 0.50% for repolarization, while KOL reported 1.36% and 0.26%, respectively. Figs 14–17 provide qualitative evidence of this performance, displaying accurate predictions across epicardial, midmyocardial, and endocardial layers.

### 3D unstructured ventricle

In the last test case considered, we solved the problem on an unstructured mesh representing a human ventricle, consisting of approximately 35k degrees of freedom. In this case, the FNO was adapted to handle irregular domains. In particular, we implemented a Non-Uniform Discrete Fourier Transform (NUDFT) extending to the 3D case the approach presented in [29]. On the other hand, KOL did not require any specific modifications to operate in this unstructured setting. Since the input is defined on an unstructured grid, we modified its representation accordingly. Specifically, each dataset consists of *N* samples, where each sample corresponds to a vector of size $N_{\text{nodes}}$ with binary values: a value of 1 at a given node indicates the presence of an external stimulus, while 0 represents the absence of stimulation. Given that all stimuli have the same pulse intensity and duration, the input applied current was reformulated as a matrix of dimension $N\times N_{\text{stim}}\times 3$, where $N_{\text{stim}}$ represents the maximum number of stimulated nodes across all samples, with padding applied where necessary. The numerical solutions for the activation and the repolarization times were structured as tensors of size $N\times N_{\text{nodes}}\times 3$, capturing the time evolution of the solution on the unstructured mesh.

The results in Table 4 provide insights into the performance of FNO and KOL on this complex domain. The FNO architecture employed consisted of Fourier layers with four Fourier modes in each spatial direction (*x*, *y*, and *z*), leading to trainable parameters ranging between 10.8M and 11.3M. We applied a reduceOnPlateau learning rate policy, as it consistently yielded better predictions in previous tests by dynamically adjusting the learning rate when the test loss plateaued. The architecture’s depth and width also influenced performance. Notably, for *N* = 2000, increasing the model width from 2 to 16 substantially improved accuracy, reducing the test error from $1.38\times 10^{-1}$ to $5.86\times 10^{-2}$ (cf. Table D in S1 File). However, beyond a certain point, further increases in width did not yield significant improvements, with test errors fluctuating for widths above 32. Similarly, deeper architectures (*L*>1) did not always lead to better performance, indicating that an optimal hyperparameters selection is crucial for balancing expressivity and generalization. In Table 4 we reported the most accurate performances of FNOs obtained considering *L* = 1, width=16 (acti) and *L* = 3, width=32 (repo).

**Table 4:** Performance comparison of FNO and KOL on 3D unstructured datasets (cfr. Table D in S1 File for the performance comparison of FNO architectures with different layers and widths).

	Dataset (size)	Test Error	GPU Memory	Training Time	Test Time	Pearson (test)
FNO with lr policy reduceOnPlateau	acti (2000)	5.86E-02 ± 2.98E-03	6.13GB	80 min	8.4E-03 sec	5.6E-03
	repo (2000)	2.42E-02 ± 1.00E-02	6.15GB	84 min	9.6E-03 sec	2.6E-03
	**Dataset (size)**	**Test Error**	**CPU Memory**	**Training Time**	**Test Time**	**Pearson (test)**
KOL with iq4 kernel	acti (2000)	1.36E-02	1.37GB	4 min	4.7E-02 sec	6.6E-05
	repo (2000)	2.53E-03	1.34GB	4 min	4.8E-02 sec	6.1E-05

**Fig 18:**
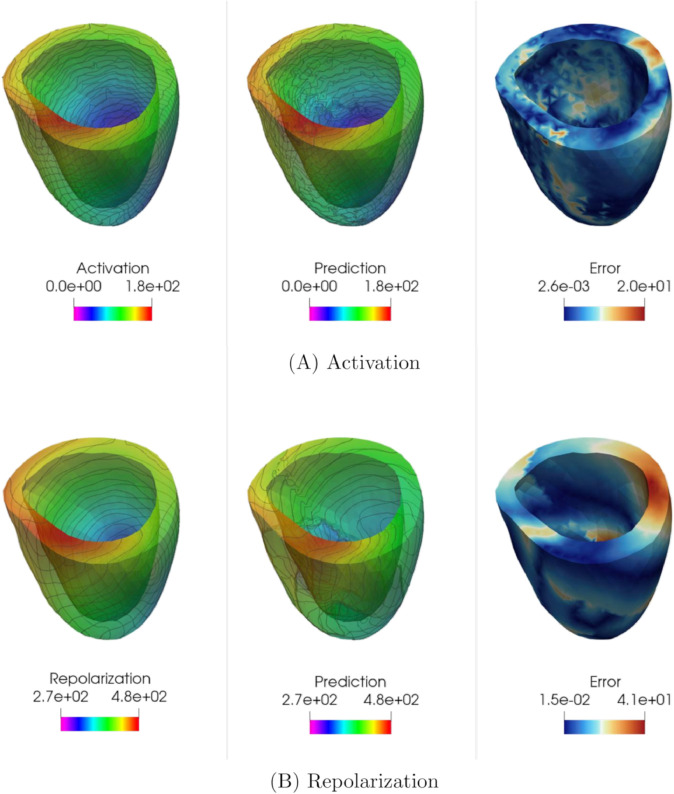
Example of FNO predictions for the 3D unstructured case: (A) Activation times (acti 2000), (B) Repolarization times (acti 2000). Colorbars indicate time in milliseconds (ms).

**Fig 19:**
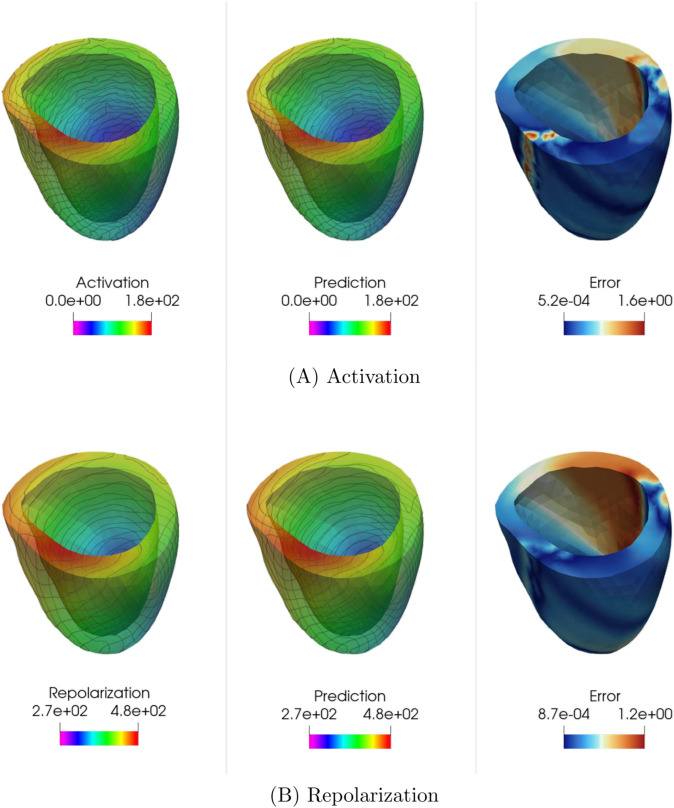
Example of KOL predictions for the 3D unstructured case: (A) Activation times (acti 2000), (B) Repolarization times (acti 2000). Colorbars indicate time in milliseconds (ms).

Also in this case, KOL (endowed with iq4 kernel) significantly outperforms FNO in terms of test error, achieving a test error as low as $2.53\times 10^{-3}$ on the repolarization dataset for *N* = 2000, compared to FNO’s $2.42\times 10^{-2}$. The difference is even more pronounced for the activation dataset, where KOL attains a test error of 1.36 × 10^−2^, while FNO’s best result remains at 5.86 × 10^−2^. Additionally, KOL exhibits lower Pearson dissimilarity values, indicating a better linear correlation between the ground truth test data and the corresponding predictions. Solutions for a specific test case, for both activation and repolarization, are shown in Figs 18 and 19. The absolute error plots highlight the lower error achieved by KOL, with a maximum absolute error of 4 ms, compared to larger regions reaching approximately 8.6 ms in the FNO case. However, both architectures successfully captured the qualitative distribution of activation and repolarization times.

Computationally, FNO has an advantage in terms of inference time. A single prediction with FNO on the larger dataset, consisting of 2000 samples with 1600 for training and 400 for testing, takes approximately 8.5 ms, whereas KOL requires 56 ms for the same task. Both methods outperform the solution of a single Monodomain model implemented using the PETSc library [4] on a node equipped with an Intel(R) Xeon(R) Silver 4316 CPU @ 2.30GHz and two Nvidia H100 GPUs, which requires about 4 minutes on 2 cores with GPU acceleration. In order to have a fair comparison from a user’s point of view, we have performed the single prediction timing tests on an laptop equipped with an Apple M1 Pro chip, while the trainings have been performed on a machine equipped with an NVIDIA Quadro RTX 5000 GPU for FNO, while on an Intel i7 machine for KOL.

Furthermore, in this case training KOL is significantly faster than FNO, requiring only a few minutes compared to FNO’s training time, which ranges from 58 to 84 minutes depending on the configuration. KOL is also much more memory efficient, using just over 1GB of CPU memory, compared to FNO’s GPU memory consumption of around 6.1GB. These results suggest that KOL is lighter, faster and more accurate than FNO, even though we expect the latter to be more time-efficient when predicting a large number of occurrences is required.

## Conclusions

In this study, we developed and formalized operator learning approaches to reconstruct activation and repolarization times in cardiac tissue, given an input activation region corresponding to electrically stimulated cells. This problem is particularly significant for clinicians, as utilizing computational architectures for patient-specific simulations can improve clinical decision-making. Although activation and repolarization times can be derived from PDE-based models, simulating these processes entails solving large-scale systems, resulting in high computational costs. The time savings offered by surrogate models, compared to high-fidelity PDE-based approaches, become particularly evident in multi-query applications which require many evaluations of the same model with slight modifications of some quantities of interest, such as inverse problems, parameter estimation, and uncertainty quantification.

For this purpose, we adapted and evaluated two operator learning strategies: Fourier Neural Operators (FNO), based on the convolution theorem for the Fourier transform, and Kernel Operator Learning (KOL), based on kernel regression. These trained operator learning techniques yield accurate and computationally efficient approximations of the target maps when evaluated on new samples. Notably, in the repolarization case, we successfully approximated an operator map for which no corresponding PDE model is currently available. Training data were generated by solving the Monodomain model with spatial randomly distributed pulses with different ionic models using finite element method on 2D and 3D structured meshes, as well as on a physiologically realistic left ventricle. Both methods proved robust and accurate performance (with errors generally below 1%) while significantly reducing computational costs compared to classical FEM-based simulations in a high number of evaluations. Additionally, a systematic sensitivity analysis was conducted for the 2D case to assess hyperparameter dependence of both architectures.

Our numerical experiments demonstrate that KOL outperforms FNO in terms of accuracy and training time, even in the challenging case of 3D unstructured meshes. However, the computational gains of KOL are partially offset by the significant cost associated with kernel selection, which may pose a limitation. This latter problem may be mitigated by choosing the kernel adaptively, *e.g.* relying on the so called parametric Kernel Flows approaches [36]. Additionally, our results validate the feasibility of applying FNO to unstructured cardiac meshes, provided that suitable architectural modifications are implemented to accommodate non-uniform data structures. Finally, while KOL offers superior accuracy, it comes at the expense of an increased inference time compared to the FNO counterpart. Hence, FNO can be the eligible choice for very high number of validation data.

Future work will leverage on the proposed models, which have proven to be fast and accurate surrogate for forward simulations, for inverse problem applications which are extremely relevant in computational cardiology. In particular, a long-term goal is to enable the reconstruction of the likely site of excitation origin based on observed activation maps obtained from electroanatomical mapping or ECG imaging, such as in cases of focal arrhythmias.

Despite the promising potential of KOL and FNO for forward EP problems, a major challenge remains their extension to diverse patient-specific geometries, which will require substantially larger datasets to account for intra-sample variability during training and also test. Model generalization across geometries will be the subject of future work. One possible direction consists in exploring Universal Solution Manifold Networks (USM-Nets) [41], which are designed to learn mappings over families of PDE solutions defined on different domains. Another viable route is the integration of data assimilation techniques [40] to personalize or adapt pretrained operator models using sparse or partial observations from individual patients.

Building on the flexibility of the proposed operator learning framework, future work will explore the incorporation of additional input parameters to better represent both structural and electrophysiological heterogeneity of the cardiac tissue. In particular, we plan to encode spatially localized features, such as ischemic regions or zones of reduced conductivity, together with parameters describing inter-individual variability in ionic kinetics and electrophysiological remodeling. This extension will enable the surrogate models to generalize across a broader spectrum of physiological and pathological conditions, including drug-induced alterations, abnormal restitution dynamics, and disease-related propagation impairments. However, realizing this extension will require the generation or collection of sufficiently large and diverse training datasets that include both physiological and pathological samples, in order to ensure proper generalization and to prevent overfitting on specific configurations.

## Data collection and software

Data employed for the numerical tests presented in this work are available at https://zenodo.org/records/16913206, whilst the software is available at https://github.com/edoardo100/timeactML.git.

## Supporting information

S1 File Supplementary material. File containing all the supplementary mathematical methods, tables and figures. (PDF)
