# Supplementary material for: Learning cardiac activation and repolarization times with operator learning
Source: PLoS Comput Biol. 2026 Jan 27;22(1):e1013920. doi: 10.1371/journal.pcbi.1013920 (PMC12858077; doi:10.1371/journal.pcbi.1013920)
Supplement: S1 File — (PDF) [file pcbi.1013920.s001.pdf]

Learning cardiac activation and repolarization times with operator learning: Supporting Information

Giovanni Ziarelli[1‡, *], Edoardo Centofanti[2‡], Nicola Parolini[3], Simone Scacchi[1], Marco Verani[3], Luca F. Pavarino[2]

1 Dipartimento di Matematica, Università di Milano, Milano, Italy
2 Dipartimento di Matematica, Università di Pavia, Pavia, Italy
3 MOX Laboratory - Dipartimento di Matematica, Politecnico di Milano, Milano, Italy

Text A: Fourier Neural Operators

Fourier Neural Operators (FNOs) [4] are operator learning schemes based on the Neural Operator paradigm [3].

Definition 1 (Neural Operator)**.** *Assuming the setting introduced in Problem 1, we define the Neural Operator as the architecture $\hat{\mathcal{G}}_\theta : \mathbb{R}^n \to \mathbb{R}^m$ with the following structure:*

$$\hat{\mathcal{G}}_\theta := Q \circ \sigma_T \left(W_{T-1} + K_{T-1} + b_{T-1}\right) \circ \cdots \circ \sigma_1 \left(W_0 + K_0 + b_0\right) \circ P \tag{1}$$

where each inner operator represents a layer which maps the t-th hidden representation a_t to the next one a_{t+1}, for $t = 0 \dots T-1$, following the scheme reported in Fig. 2. More in detail, a Neural Operator is composed as follows:

- *$P : \mathbb{R}^n \to \mathbb{R}^{d_{a_0}}$, a lifting operator, namely a pointwise function mapping the observed input to its first hidden representation, i.e. $\{\phi(u) : \mathcal{U} \to \mathbb{R}^n\} \mapsto \{a_0 : \Omega_0 \subset \mathbb{R}^{d_0} \to \mathbb{R}^{d_{a_0}}\}$, with $d_{a_0} > d_0$. This operation is performed by a fully local operator, and its action is pointwise, namely $(P(u))(x) = P(u(x))$ for $x \in \Omega$;*
- *A composition of operators $\{a_t : \Omega_t \to \mathbb{R}^{d_{a_t}}\} \to \{a_{t+1} : \Omega_{t+1} \to \mathbb{R}^{d_{a_{t+1}}}\}$, for $t = 0, \dots, T-1$, each one defined as the sum of a local linear operator $W_t \in \mathbb{R}^{d_{a_{t+1}} \times d_{a_t}}$, a non-local integral kernel operator K_t, and a bias function $b_t : \Omega_{t+1} \to \mathbb{R}^{d_{a_{t+1}}}$. This sum is then composed with a fixed pointwise nonlinearity σ_t, called activation function, namely $(\sigma_t(a_{t+1}))(x) = \sigma(a_{t+1}(x))$ for any $x \in \Omega_{t+1}$. We set $\Omega_0 = \Omega$ and $\Omega_T = \Omega'$, and assume that each $\Omega_t \subset \mathbb{R}^{d_t}$ is a bounded domain.*
- *$Q : \mathbb{R}^{d_{a_T}} \to \mathbb{R}^m$, a projection operator, namely a pointwise function, mapping the last hidden representation $\{a_T : \Omega' \to \mathbb{R}^{d_{a_T}}\} \mapsto \{\varphi(u) : \mathcal{U} \to \mathbb{R}^m\}$ to the observed output function. Since $d_{a_t} > m$, this is a projection step performed by a fully local operator, namely $(Q(a_T))(x) = Q(a_T(x))$ for every $x \in \Omega'$.*

Input and output dimensions $d_{a_0}, \cdots, d_{a_T}$ as well as the domains of definition $\Omega_1, \cdots, \Omega_{T-1}$ are hyperparameters of the architecture.

In this work, we follow the first definition for the integral kernel operators proposed in [3]. As a technical remark, we denote as $C(\Omega_{t+1} \times \Omega_t, \mathbb{R}^{d_{a_{t+1}} \times d_{a_t}})$ the Banach space

of continuous functions from $\Omega_{t+1} \times \Omega_t$ to $\mathbb{R}^{d_{a_{t+1}} \times d_{a_t}}$ equipped with the sup-norm $||g||_\infty := \sup\{|g(\tau)| : \tau \in \Omega_{t+1} \times \Omega_t\}$, $g \in C(\Omega_{t+1} \times \Omega_t, \mathbb{R}^{d_{a_{t+1}} \times d_{a_t}})$.

In this context, given $\kappa^{(t)} \in C(\Omega_{t+1} \times \Omega_t; \mathbb{R}^{d_{a_{t+1}} \times d_{a_t}})$ such that $\kappa^{(t)}(x, y) = \kappa^{(t)}(x - y)$, for all $(x, y) \in \Omega_{t+1} \times \Omega_t$, the integral kernel operator K_t is defined as

$$(K_t(a_t))(x) = \int_{\Omega_t} \kappa^{(t)}(x - y) a_t(y)\, dy \quad \forall x \in \Omega. \tag{2}$$

At this point, we can thus make precise the single hidden layer update rule as

$$a_{t+1}(x) = \sigma_{t+1} \left(W_t a_t\,(x) + \int_{\Omega_t} \kappa^{(t)}(x, y) a_t(y)\, d\nu_t(y) + b_t(x) \right) \qquad \forall x \in \Omega_{t+1}. \tag{3}$$

From this general structure for the Neural Operator, one can derive the FNO by discretizing the integral kernel (2) in the Fourier space. The key feature of the FNO is that the integral kernel is parameterized in the Fourier space. Therefore, in the following we recall the Fourier transform $\mathcal{F} : L^2(\Omega, \mathbb{C}^n) \to \ell^2(\mathbb{Z}^d, \mathbb{C}^n)$ and anti-transform $\mathcal{F}^{-1}$. Given $a \in L^2(\Omega, \mathbb{C}^n)$ and $\hat{a} \in \ell^2(\mathbb{Z}^d, \mathbb{C}^n)$, we define

$$\begin{aligned}
&(\mathcal{F}a)_j\,(k) = \hat{a}_j(k) = \langle a_j, \varphi_k \rangle_{L^2(\Omega, \mathbb{C}^n)}, \qquad j \in \{1, \cdots, n\}, \quad k \in \mathbb{Z}^d, \\
&\left(\mathcal{F}^{-1}\hat{a}\right)_j(x) = \sum_{k \in \mathbb{Z}^d} \hat{a}_j(k) \varphi_k^{-1}(x), \qquad j \in \{1, \cdots, n\}, \quad x \in \Omega \\
&\varphi_k(x) := e^{-2\pi i k \cdot x}, \qquad x \in \Omega.
\end{aligned} \tag{4}$$

For the FNO, the domain considered for each layer is the periodic torus $\Omega_t = \mathbb{T}^d = [0, 2\pi]^d$, although for a general input it is sufficient to take its periodic extension rescaled in $[0, 2\pi]^d$.

The integral kernel $\kappa^{(t)}$ in (2) is a function parameterized by some parameters θ_t belonging to a suitable space $\Theta_t \subset \mathbb{R}^{d_{a_{t+1}} \times d_{a_t}}$. Thus we write $\kappa^{(t)} = \kappa^{(t)}_{\theta_t}$. From (2), we set $\kappa^{(t)}_{\theta_t}(x, y) = \kappa^{(t)}_{\theta_t}(x - y)$ and we apply the convolution theorem for the Fourier transform,

$$(K_t(a_t))(x) = \int_{\Omega} \kappa^{(t)}_{\theta_t}(x - y) a_t(y)\, dy = \mathcal{F}^{-1}\left(\mathcal{F}\left(\kappa^{(t)}_{\theta_t}\right) \cdot \mathcal{F}(a_t) \right)(x), \qquad \forall x \in \Omega_t, \tag{5}$$

where the dot operation is defined as

$$\mathcal{F}\left(\kappa^{(t)}_{\theta_t}\right) \cdot \mathcal{F}(a_t) := \sum_{k \in \mathbb{Z}} \mathcal{F}\left(\kappa^{(t)}_{\theta_t}\right)(k) \cdot \mathcal{F}(a_t)(k) = \sum_{k \in \mathbb{Z}} \hat{\kappa}^{(t)}_{\theta_t}(k) \cdot \hat{v}_t(k). \tag{6}$$

Considering $k \in \mathbb{Z}^d$ fixed as frequency mode, we have that $\mathcal{F}\left(\kappa^{(t)}_{\theta_t}\right)(k) \in \mathbb{C}^{d_{a_{t+1}} \times d_{a_t}}$ and $\mathcal{F}(a_t)(k) \in \mathbb{C}^{d_{a_t}}$. Hence, $\kappa^{(t)}_{\theta_t}$ can be parameterized directly by its Fourier coefficients and (5) reads as:

$$(K_t(a_t))(x) = \mathcal{F}^{-1}\left(R_{\theta_t} \cdot \mathcal{F}(a_t)\right)(x), \qquad \forall x \in \Omega_t, \tag{7}$$

where $R_{\theta_t} = \hat{\kappa}^{(t)}_{\theta_t}(k)$ for k fixed. Fig. 2 represents the schematic FNO architecture considered in this work.

Numerically, the FNO needs to be approximated from continuous space to discrete ones for dealing with finite-dimensional parameterizations. We call pseudo(Ψ)-Fourier Neural Operator (Ψ-FNO) the approximated architecture, following [2]. In this case, the domain Ω_t is discretized with $J \in \mathbb{N}$ points, and therefore a_t can be treated as a tensor

in $\mathbb{C}^{J\times d_{a_t}}$. Since integrals cannot be calculated exactly, this leads to considering the Fourier series truncated at a maximal mode $k_{\max}$, such that

$$k_{\max} = \left|\{k \in \mathbb{Z}^d \ : \ |k_j| \le k_{\max,j}, \text{ for } j = 1, \dots, d\}\right|.$$

In practical implementations, the Fourier transform is replaced by the Fast Fourier Transform (FFT), and the weight tensor R_{θ_t} is parameterized as a complex-valued tensor $R_{\theta_t} \in \mathbb{C}^{k_{\max}\times d_{a_{t+1}}\times d_{a_t}}$ for $i = 1, \dots, L$. For simplicity, we denote R_{θ_t} with R. Finally, we note that if a_t is real-valued, we can enforce conjugate symmetry in tensor R for imposing a_{t+1} to be real-valued, namely $R(-k)_{j,l} = \overline{R}(k)_{j,l}$.

Text B: Kernel functions for KOL

In this work we undergo a sensitivity analysis for Kernel Operator Learning by training the operator learning schemes with three different families of kernel.

- **Radial Basis Functions (RBF) kernel**: $S(A_1, A_2) = e^{-\frac{\|\mathbf{c}_{A_1} - \mathbf{c}_{A_2}\|_2^2}{2\sigma^2}}$, where $\mathbf{c}_{A_*}$ represents the vector of coordinates of the centroid of non-zero elements of discrete observations in A_*. It has the interpretation of a similarity measure and it decreases as long as the distance between points increases. In the case of RBF kernel, Kernel Operator Learning has an explicit connection with Gaussian Processes (GP) for regression tasks (see, *e.g.*, [5]).

- **Neural Tangent Kernel (NTK)**: Given a neural network regressor $f(x;\theta)$ of depth d_{nn}, width l_{nn} and activation function σ_{nn}, with θ_{nn} denoting the vector collecting all weights and biases, we define the family of finite-width Neural Tangent Kernels $\{S\}_{\tau>0} : \mathbb{R}^n \times \mathbb{R}^n \to \mathbb{R}$ as

$$S_\tau(A_i, A_j) := \langle \partial_{\theta_{\text{nn}}} f(A_j; \theta_{\text{nn}}(\tau)),\, \partial_{\theta_{\text{nn}}} f(A_i; \theta_{\text{nn}}(\tau))\rangle, \tag{8}$$

  where τ represents a fictitious iteration time. It has been proven that, if the initialization of the weights follows the so-called NTK initialization [1], in the infinite-width limit each element in the sequence $\{S_\tau\}_\tau$ converges in probability to a stationary kernel independently on τ, *i.e.*

$$S_\tau(A_i, A_j) \underset{\mathbb{P}}{\to} S(A_i, A_j),\ \forall\, \tau > 0,\ \forall\, A_i, A_j. \tag{9}$$

  Hence, the family of NTKs strictly depends on two parameters: activation function and depth of the associated neural network. In this paper, with NTK we refer to the infinite-width limit kernel function.

- **Euclidean distance between centroids (IQ)**: $S(A_1, A_2) = \frac{1}{\sqrt{\sigma_1 \|\mathbf{c}_{A_1} - \mathbf{c}_{A_2}\| + \sigma_2}}$, where, even in this case, $\mathbf{c}_{A_*}$ is the vector of coordinates of the centroid of non-zero elements of discrete observations in A_*. This kernel function is driven by the physics of the problem at stake, estimating the distance between centroids of the activation region in Euclidean metrics.

Each of the kernel functions considered depends on specific hyperparameters that need to be tuned for the given physical application, *e.g.* the variance for RBF kernels, the width and depth of NTK, and the constants σ_1, σ_2 for the IQ kernel. A table summarizing the tuned hyperparameters for each KOL scheme used in the 2D sensitivity analysis is provided in the Supporting Information (ST5).

Additional Tables

	Test Error	Dataset (size)	lr policy	GPU Memory	Training Time
FNO	3.33E-03 ± 8.41E-05	acti (2000)	reduceOnPlateau	5.62GB	48 min
	2.66E-03 ± 2.27E-04	acti (3000)		5.79GB	72 min
	3.33E-03 ± 3.13E-04	acti rot (2000)		5.62GB	48 min
	3.64E-03 ± 9.13E-04	repo (2000)		5.62GB	48 min
	3.13E-03 ± 2.33E-04	repo (3000)		5.79GB	72 min
	3.53E-03 ± 3.63E-04	repo rot (2000)		5.62GB	48 min
	3.80E-03 ± 1.45E-04	acti (2000)	None	5.69GB	48 min
	2.82E-03 ± 2.28E-04	acti (3000)		5.79GB	72 min
	3.77E-03 ± 9.65E-04	acti rot (2000)		5.62GB	48 min
	3.40E-03 ± 5.77E-05	repo (2000)		5.62GB	48 min
	3.53E-03 ± 3.32E-04	repo (3000)		5.79GB	72 min
	4.16E-03 ± 4.04E-05	repo rot (2000)		5.62GB	48 min

Table A. Performance comparison of FNO on 2D datasets.

	Test Error	Dataset (size)	Kernel (iq)	CPU Memory	Training Time
KOL	9.33E-04	`acti rot` (2000)	iq-1	0.91GB	476 sec
	9.35E-04	`acti rot` (2000)	iq-2	0.92GB	517 sec
	9.39E-04	`acti rot` (2000)	iq-3	0.91GB	517 sec
	9.34E-04	`acti rot` (2000)	iq-4	0.91GB	459 sec
	1.15E-03	`acti rot` (2000)	iq-5	0.97GB	477 sec
	1.19E-01	`acti rot` (2000)	ntk-1	0.97GB	660 sec
	1.41E-01	`acti rot` (2000)	ntk-2	0.97GB	691 sec
	1.42E-01	`acti rot` (2000)	ntk-3	0.97GB	687 sec
	1.73E-01	`acti rot` (2000)	rbf-1	0.92GB	93 sec
	1.19E-01	`acti rot` (2000)	rbf-2	0.92GB	92 sec
	1.18E-01	`acti rot` (2000)	rbf-3	0.92GB	89 sec
	1.07E-03	`acti` (2000)	iq-1	0.91GB	444 sec
	1.08E-03	`acti` (2000)	iq-2	0.91GB	451 sec
	1.09E-03	`acti` (2000)	iq-3	0.92GB	442 sec
	1.08E-03	`acti` (2000)	iq-4	0.92GB	448 sec
	1.38E-03	`acti` (2000)	iq-5	0.91GB	448 sec
	1.31E-01	`acti` (2000)	ntk-1	0.97GB	656 sec
	1.54E-01	`acti` (2000)	ntk-2	0.97GB	652 sec
	1.55E-01	`acti` (2000)	ntk-3	0.97GB	636 sec
	1.86E-01	`acti` (2000)	rbf-1	0.92GB	82 sec
	1.31E-01	`acti` (2000)	rbf-2	0.92GB	80 sec
	1.30E-01	`acti` (2000)	rbf-3	0.92GB	80 sec
	9.51E-04	`acti` (3000)	iq-1	1.15GB	613 sec
	9.53E-04	`acti` (3000)	iq-2	1.15GB	609 sec
	9.54E-04	`acti` (3000)	iq-3	1.15GB	616 sec
	9.52E-04	`acti` (3000)	iq-4	1.15GB	611 sec
	1.11E-03	`acti` (3000)	iq-5	1.15GB	611 sec
	1.28E-01	`acti` (3000)	ntk-1	1.20GB	979 sec
	1.52E-01	`acti` (3000)	ntk-2	1.20GB	980 sec
	1.51E-01	`acti` (3000)	ntk-3	1.20GB	1007 sec
	1.83E-01	`acti` (3000)	rbf-1	1.18GB	117 sec
	1.28E-01	`acti` (3000)	rbf-2	1.15GB	125 sec
	1.27E-01	`acti` (3000)	rbf-3	1.15GB	127 sec

Table B. Performance comparison of KOL on 2D datasets for reconstructing activation maps.

	Test Error	Dataset (size)	Kernel (iq)	CPU Memory	Training Time
KOL	4.69E-04	repo rot (2000)	iq-1	0.91GB	418 sec
	4.67E-04	repo rot (2000)	iq-2	0.91GB	389 sec
	4.69E-04	repo rot (2000)	iq-3	0.92GB	389 sec
	4.69E-04	repo rot (2000)	iq-4	0.91GB	393 sec
	6.72E-04	repo rot (2000)	iq-5	0.91GB	397 sec
	5.95E-02	repo rot (2000)	ntk-1	0.97GB	575 sec
	7.02E-02	repo rot (2000)	ntk-2	0.97GB	571 sec
	6.98E-02	repo rot (2000)	ntk-3	0.97GB	565 sec
	8.33E-02	repo rot (2000)	rbf-1	0.92GB	72 sec
	5.93E-02	repo rot (2000)	rbf-2	0.92GB	71 sec
	5.91E-02	repo rot (2000)	rbf-3	0.92GB	72 sec
	4.93E-04	repo (2000)	iq-1	0.91GB	408 sec
	4.92E-04	repo (2000)	iq-2	0.91GB	450 sec
	5.19E-04	repo (2000)	iq-3	0.91GB	473 sec
	4.93E-04	repo (2000)	iq-4	0.91GB	473 sec
	7.82E-04	repo (2000)	iq-5	0.92GB	414 sec
	6.49E-02	repo (2000)	ntk-1	0.97GB	595 sec
	7.64E-02	repo (2000)	ntk-2	0.97GB	632 sec
	7.61E-02	repo (2000)	ntk-3	0.97GB	622 sec
	9.01E-02	repo (2000)	rbf-1	0.92GB	74 sec
	6.47E-02	repo (2000)	rbf-2	0.92GB	76 sec
	6.44E-02	repo (2000)	rbf-3	0.92GB	90 sec
	4.74E-04	repo (3000)	iq-1	1.15GB	592 sec
	4.72E-04	repo (3000)	iq-2	1.15GB	576 sec
	4.97E-04	repo (3000)	iq-3	1.15GB	574 sec
	4.74E-04	repo (3000)	iq-4	1.15GB	582 sec
	7.19E-04	repo (3000)	iq-5	1.15GB	573 sec
	6.20E-02	repo (3000)	ntk-1	1.20GB	1064 sec
	7.39E-02	repo (3000)	ntk-2	1.20GB	1007 sec
	7.34E-02	repo (3000)	ntk-3	1.20GB	987 sec
	8.77E-02	repo (3000)	rbf-1	1.15GB	120 sec
	6.19E-02	repo (3000)	rbf-2	1.15GB	117 sec
	6.16E-02	repo (3000)	rbf-3	1.15GB	117 sec

Table C. Performance comparison of KOL on 2D datasets for reconstructing repolarization maps.

Test Error	Dataset (size)	L	width	parameters	GPU Memory	Training Time	Test Time	Pearson (test)
1.07E-01 ± 2.43E-02	acti (1000)	3	32	10.9M	6.06GB	58 min	1.0E-02 sec	3.9E-02
3.06E-01 ± 1.21E-01		4	32	10.9M	6.07GB	59 min	1.0E-02 sec	2.9E-02
1.38E-01 ± 1.76E-02	acti (2000)	1	2	10.8M	6.12GB	81 min	8.5E-03 sec	3.9E-02
8.44E-02 ± 6.27E-03		1	4	10.8M	6.12GB	80 min	8.7E-03 sec	1.4E-02
7.22E-02 ± 6.36E-04		1	8	10.8M	6.12GB	81 min	8.4E-03 sec	7.8E-03
5.86E-02 ± 2.98E-03		1	16	10.9M	6.13GB	79 min	8.7E-03 sec	5.6E-03
8.34E-02 ± 3.02E-02		3	32	11.0M	6.15GB	83 min	9.7E-03 sec	1.7E-02
8.35E-02 ± 3.10E-02		2	32	10.9M	6.14GB	82 min	9.1E-03 sec	1.5E-02
7.27E-02 ± 1.09E-02		1	32	10.9M	6.13GB	80 min	8.8E-03 sec	5.0E-03
1.10E-01 ± 1.30E-02		1	64	11.0M	6.14GB	80 min	8.6E-03 sec	2.0E-02
1.34E-01 ± 1.39E-02		1	128	11.3M	6.17GB	81 min	9.5E-03 sec	2.5E-02
2.42E-02 ± 1.00E-02	repo (2000)	3	32	10.9M	6.15GB	84 min	9.6E-03 sec	2.6E-03

Table D. Performance comparison of FNO on 3D unstructured datasets. Time single prediction test performed on a machine equipped with chip Apple M1 Pro.

Name	Kernel Type	σ	d_{nn}	Activation Function	σ_1	σ_2
iq1	IQ	/	/	/	1E-5	1E-2
iq2	IQ	/	/	/	1E-5	1E-1
iq3	IQ	/	/	/	1E-4	1E-2
iq4	IQ	/	/	/	1E-4	1E-1
iq5	IQ	/	/	/	1E-3	1E-2
rbf1	RBF	1	/	/	/	/
rbf2	RBF	10	/	/	/	/
rbf3	RBF	100	/	/	/	/
ntk1	NTK	/	3	Sigmoid	/	/
ntk2	NTK	/	4	Sigmoid	/	/
ntk3	NTK	/	3	ReLu	/	/

Table E. Nomenclature of kernel functions tested for KOL.

Additional Figures

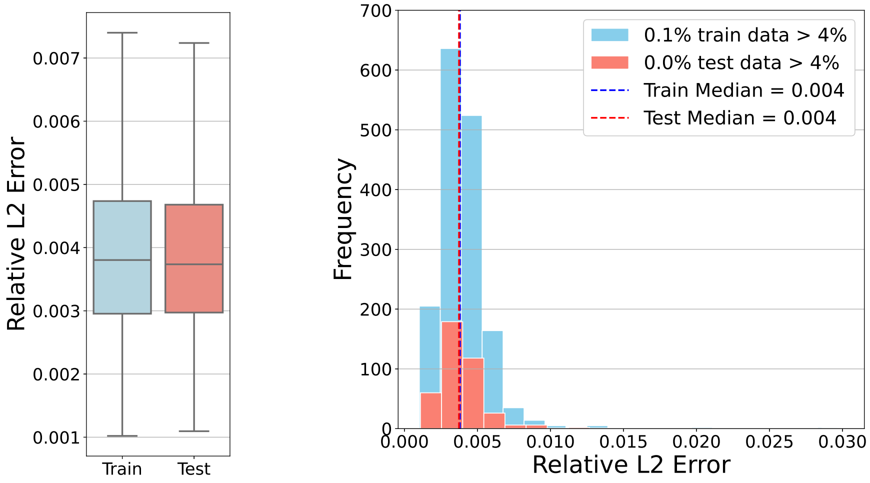

(A) FNO box plot. (B) FNO histogram.

Fig A. FNO box plot (A) and histogram (B) for the 3D dataset `repo 2000` relative to the best model trained.

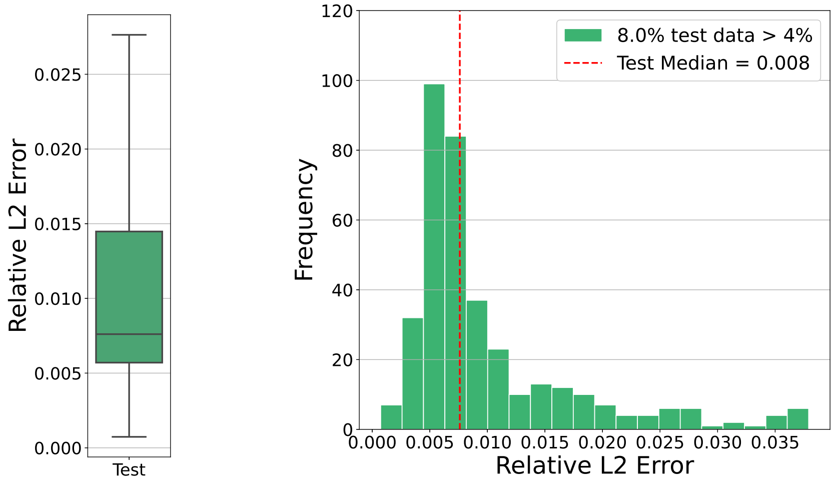

(A) KOL box plot. (B) KOL histogram.

Fig B. KOL box plot (A) and histogram (B) for 3D dataset `repo 2000` relative to the best model trained. Training results are not shown since we achieve machine precision.

References

1. Jacot A, Gabriel F, Hongler C. Neural tangent kernel: Convergence and generalization in neural networks. Adv Neural Inf Process Syst. 2018;31.

2. Kovachki N, Lanthaler S, Mishra S. On universal approximation and error bounds for Fourier neural operators. J Mach Learn Res. 2021;22(1):13237–312.

3. Kovachki N, Li Z, Liu B, Azizzadenesheli K, Bhattacharya K, Stuart A, et al. Neural operator: Learning maps between function spaces with applications to PDEs. J Mach Learn Res. 2023;24(89):1–97.

4. Li Z, Kovachki N, Azizzadenesheli K, Liu B, Bhattacharya K, Stuart A, et al. Fourier neural operator for parametric partial differential equations. arXiv preprint. 2020;arXiv:2010.08895.

5. Smola AJ, Schölkopf B, Müller KR. The connection between regularization operators and support vector kernels. Neural Netw. 1998;11(4):637–49.
